# Retinal degeneration in progressive supranuclear palsy measured by optical coherence tomography and scanning laser polarimetry

**DOI:** 10.1038/s41598-017-05575-8

**Published:** 2017-07-13

**Authors:** Birthe Stemplewitz, Robert Kromer, Eik Vettorazzi, Ute Hidding, Andreas Frings, Carsten Buhmann

**Affiliations:** 1 Department of Ophthalmology, University Medical Center Hamburg-Eppendorf, Martinistrasse 52, 20246 Hamburg, Germany; 2 Department of Medical Biometry and Epidemiology, University Medical Center Hamburg-Eppendorf, Martinistrasse 52, 20246 Hamburg, Germany; 3 Department of Neurology, University Medical Center Hamburg-Eppendorf, Martinistrasse 52, 20246 Hamburg, Germany

## Abstract

This cross-sectional study compared the retinal morphology between patients with progressive supranuclear palsy (PSP) and healthy controls. (The retinal nerve fiber layer (RNFL) around the optic disc and the retina in the macular area of 22 PSP patients and 151 controls were investigated by spectral domain optical coherence tomography (SD-OCT). Additionally, the RNFL and the nerve fiber index (NFI) were measured by scanning laser polarimetry (SLP). Results of RNFL measurements with SD-OCT and SLP were compared to assess diagnostic discriminatory power. Applying OCT, PSP patients showed a smaller RNFL thickness in the inferior nasal and inferior temporal areas. The macular volume and the thickness of the majority of macular sectors were reduced compared to controls. SLP data showed a thinner RNFL thickness and an increase in the NFI in PSP patients. Sensitivity and specificity to discriminate PSP patients from controls were higher applying SLP than SD-OCT. Retinal changes did not correlate with disease duration or severity in any OCT or SLP measurement. PSP seems to be associated with reduced thickness and volume of the macula and reduction of the RNFL, independent of disease duration or severity. Retinal imaging with SD-OCT and SLP might become an additional tool in PSP diagnosis.

## Introduction

Progressive supranuclear palsy (PSP) is an atypical parkinsonian syndrome (APS) with progressive neurodegeneration within the central nervous system (CNS) and a fatal clinical disease course. In contrast to idiopathic (typical) Parkinson disease (PD) as synucleinopathy, it belongs to the tauopathies, has much more widespread central cell degeneration than PD, and affects subcortical and cortical areas. PSP is clinically characterized by an akinetic-rigid syndrome and also “atypical” symptoms, including early falls, oculomotor disturbances, and fronto-executive impairment. The progressive and severe oculomotor dysfunction with the characteristic vertical gaze palsy is caused by central nervous dysfunction and was name-giving for the disease. Although impairment of visual acuity, color vision or contrast^1^, and diplopia^2^ can also be found in patients with PD, visual disturbance in patients with PSP is much more profound and PSP patients frequently suffer from reading difficulties, indistinct vision, and diplopia, which are likely caused mainly by the progressive oculomotor dysfunction^3^. However, findings of visuospatial deficits^4^ or detected abnormal visual event-related potentials in patients with PSP^5^ suggest additional dysfunction of central processing mechanisms and potential changes of the retina as part of the pathology.

In recent years, optical coherence tomography (OCT), a non-invasive and contact-less method, has been increasingly used for *in vivo* imaging of the retina. OCT allows, amongst others, quantitative measurements of the retinal nerve fiber layer (RNFL) thickness and macula thickness.

In PD, changes in retinal morphology have been investigated using time or spectral domain (SD) OCT, which has shown thinning of the RNFL^6, 7^ either peripapillary^8^, in different retinal areas^9^ or in the fovea^10–13^ and also thinning of the macula^7, 14^ whereas others found no differences of the RNFL^13, 15^. Furthermore, alterations in the retinal vascular architecture of PD patients have recently been detected^16^. Another recently published study using OCT has described distinct specific disease-related retinal changes, also in atypical parkinsonian syndromes^17^, suggesting specific disease-related retinal changes in patients with different APS, including PSP.

In this study, we aimed to evaluate changes in the retinal morphology in PSP patients using SD-OCT. In addition, the RNFL was determined using scanning laser polarimetry (SLP) and results were compared with the OCT data. SLP allows detection of degenerative changes of the RNFL. The device is well established in the diagnostics of glaucoma patients^18, 19^ and has been applied recently in PD patients^7^. To our knowledge, SLP data of PSP patients has not been previously published.

We hypothesize that alterations in retinal morphology are present in PSP, might contribute to vision-related symptoms in this neurodegenerative APS and can be assessed applying SD-OCT and SLP.

## Results

Table 1 shows the characteristics of the 22 PSP patients. Patients had a mean age of 66.2 ± 6.5 (range 49–73) years. The mean disease duration was 4.3 (range 0.5–8) years and mean PSP rating scale (PSP-RS, set out by the National Institute for Neurological Disorders and Society for PSP (NINDS-SPSP), see Methods^20^) was 41.6 ± 15.0 (range 16–68). Twelve patients were male. The mean age of the 151 healthy controls was 58.2 ± 12.7 (range 21–79) years; 64 were male.

**Table 1:** Characteristics of PSP patients (n = 22) including demographic and diagnostic data.

Case	Gender	Age [years]	Disease duration [years]	PSP-Subtyp	Certainty of diagnosis	Score of PSP Rating Scale							MRT	FP-CIT SPECT	Other diagnostic Imaging	Clinical response to L-dopa use	Clinical response to long term L-dopa challenge
						History	Mentation	Bulbar	Ocular	Limb	Gait	Total					
1	female	49	5	PSP-RS	possible	6	1	3	8	5	8	31	x	x		−	−
2	female	66	4	PSP-P	probable	12	6	4	14	8	14	58	x	x	FDG-PET	−	−
3	male	69	4	PSP-RS	probable	8	5	2	9	4	10	38	x	x	MIBG-Spect	−	−
4	female	61	5	PSP-RS	possible	1	2	1	9	2	7	22	x	x	IBZM-Spect	NA	−
5	male	72	4	PSP-RS	probable	8	6	5	5	4	12	40	x			−	−
6	female	70	3	PSP-RS	probable	12	3	5	13	4	10	47	x		FDG-PET	NA	−
7	male	66	7	PSP-P	possible	5	0	1	1	4	5	16	x	x		NA	−
8	male	71	3	PSP-RS	probable	13	7	6	14	10	18	68	x			+	−
9	female	73	4	PSP-FTDP	probable	3	4	0	3	2	6	18	x	x		NA	−
10	female	71	6	PSP-RS	probable	15	6	5	13	7	18	64	x	x		NA	−
11	male	58	5	PSP-P	possible	10	1	4	9	5	7	36		x	IBZM-Spect	−	−
12	male	70	9	PSP-P	possible	6	3	1	11	5	9	34	x	x		NA	−
13	male	67	4	PSP-RS	probable	10	8	6	14	4	12	54	x	x	MIBG-Spect	NA	−
14	male	71	6	PSP-P	possible	9	2	2	11	10	17	51	x			−	−
15	female	55	4	PSP-RS	probable	17	11	4	11	8	17	68	x			−	−
16	female	68	3	PSP-P	possible	16	2	3	7	5	9	42	x		MIBG-Spect	+	+
17	female	72	6	PSP-RS	possible	9	1	3	12	4	12	41	x			+	−
18	male	73	3	PSP-RS	possible	12	1	4	7	6	12	42	x		IBZM-Spect	NA	−
19	male	67	5	PSP-CBS	possible	14	8	2	5	9	6	45	x	x	MIBG-Spect IBZM-Spect	NA	−
20	male	61	2	PSP-RS	probable	12	1	4	13	4	10	44	x	x		NA	−
21	male	69	1	PSP-RS	possible	9	7	3	8	5	4	36	x			−	−
22	female	58	1	PSP-RS	probable	6	2	0	8	1	4	21	x			NA	−

“Disease duration [years]” was calculated from the date of the first diagnosis to the date of neurological assessment.

The diagnosis of “clinically possible” or “clinically likely” PSP was made according to the clinical criteria for the diagnosis of progressive supranuclear palsy National Institute for Neurological Disorders and Society for PSP (NINDS-SPSP).

PSP-RS: Richardson subtype of PSP, PSP-P: Parkinson subtype of PSP, PSP-FTDP: behavioral variant of PSP, PSP-CBS: Overlap syndrome of PSP with cortico-basal syndrome. Cases with application of MRI or FP-CIT-SPECT are labeled with “x”. Short term effect of L-dopa challenge was labeled with “+” in case of a positive responsev ≥30% on the UPDRS III scale and “−’’ in case of a response less than 30%. Long term effect of L-dopa use (duration more than 4 weeks) was labeled with “+” if patients and physician had the impression of at least some clinical relevant effect, otherwise response was labeled with “−”. NA = not applicable.

### Basic clinical ophthalmological measurements

Visual acuity (logMAR) was significantly worse in the PSP group [0.104 ± 0.013 logMAR versus 0.011 ± 0.005 logMAR in the control group, p < 0.001 (average ± standard error)]. The intraocular pressure did not differ significantly (15.8 ± 0.6 mmHg in the PSP group and 15.1 ± 0.3 mmHg in the control group, p = 0.3), nor did the spherical refraction (0.8 ± 0.4 dpt PSP group and 0.3 ± 0.2 dpt control group, p = 0.2), indicating similar axial lengths in both groups. These parameters did not correlate with either disease duration or PSP-RS total score in the PSP group.

### Optical coherence tomography (OCT)

**Figure 1:**
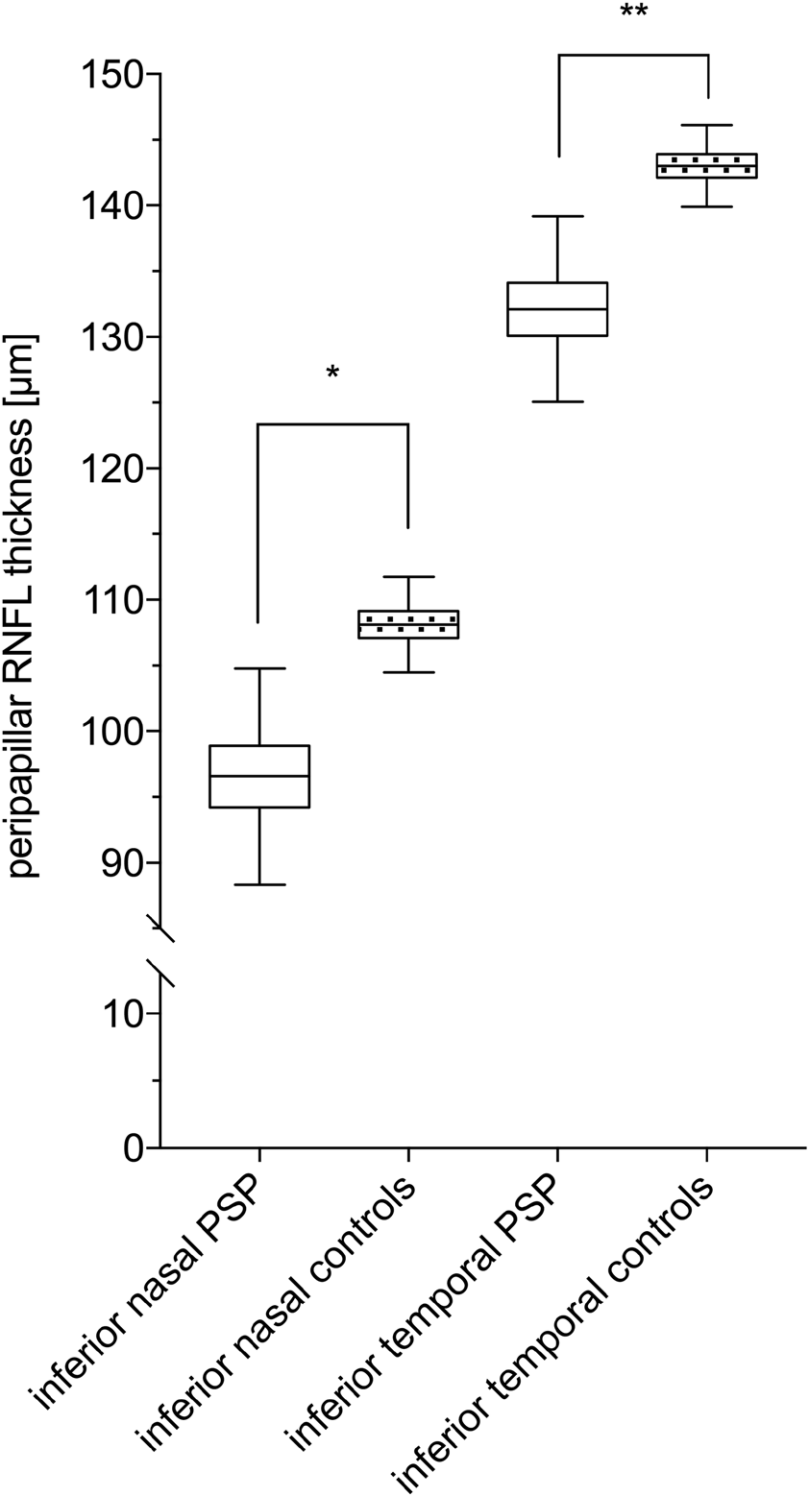
Peripapillary RNFL thickness measured by OCT. Significantly different sectors around the optic nerve head are shown. ns: not significant p > 0.5, *p ≤ 0.5, **p ≤ 0.01, ***p ≤ 0.001.

OCT RNFL data was available in 124 controls and 21 PSP patients. Significantly thinner RNFL measurements were found in the inferior nasal area [results adjusted for age and sex: 96.6 ± 4.5 μm versus 108.1 ± 2.0 μm, p = 0.02 (mean ± standard error)] and in the inferior temporal area (132.1 ± 3.9 μm versus 143.0 ± .1.7 μm, p = 0.01) around the optic nerve head (Table 2 and Fig. 1). No correlation with disease duration or the PSP rating scale was detectable.

**Table 2:** OCT RNFL measurements around the optic disc of PSP patients and control probands (mean ± standard error, corrected for age and sex).

RNFL data	PSP patients	Controls	p-values
Average RNFL thickness (µm)	93.9 ± 2.0	96.8 ± 0.8	0.20
Papillomacular bundle thickness (µm)	54.2 ± 1.9	54.5 ± 0.8	0.88
Nasal thickness (µm)	73.6 ± 2.9	74.1 ± 1.2	0.87
Nasal-superior thickness (µm)	108.3 ± 3.9	100.8 ± 1.7	0.08*
Nasal-inferior thickness (µm)	96.6 ± 4.5	108.1 ± 2.0	0.02*
Temporal thickness (µm)	71.5 ± 2.5	70.2 ± 1.1	0.61
Temporal-superior thickness (µm)	136.0 ± 3.8	133.2 ± 1.7	0.52
Temporal-inferior thickness (µm)	132.1± 3.9	143.0 ± 1.7	0.01*

P-values with significant differences are marked with *.

**Figure 2:**
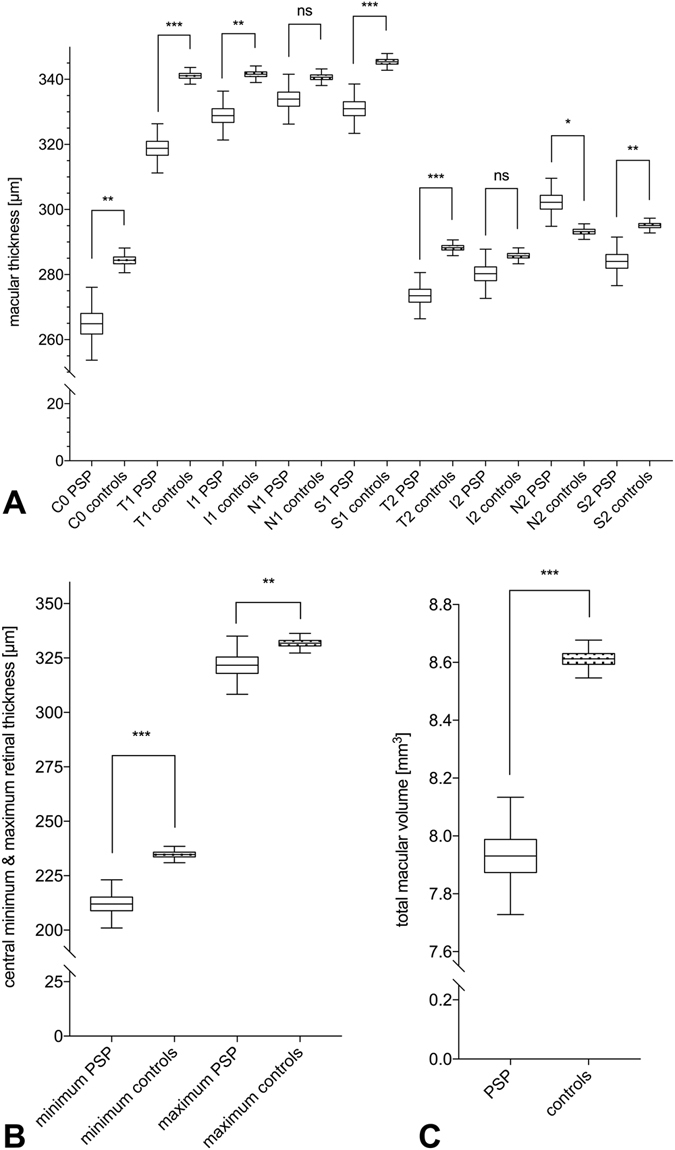
Macular data measured by OCT. (**A**) Macular thickness in all eight sectors of PSP patients and controls are shown. (**B**) Central minimum and maximum retinal thickness within the macular area are pictured. (**C**) The total macular volume in mm^3^ of PSP patients and controls is shown. ns: not significant p > 0.5, *p ≤ 0.5, **p ≤ 0.01, ***p ≤ 0.001.

The macular scans also showed statistical differences: The total macular volume (7.9 ± 0.1 mm^3^ versus 8.6 ± 0.03 mm^3^, p < 0.0001) and the central minimum (212.4 ± 5.8 μm versus 234.0 ± 1.0 μm, p = 0.001) of the PSP patients were significantly smaller compared to controls, whereas the central maximum was not significantly altered (321.8 ± 5.7 μm versus 330.6 ± 1.9 μm, p = 0.1) (Table 3 and Fig. 2B and C).

**Table 3:** OCT macular data of PSP patients and control probands (mean ± standard error, corrected for age and sex).

Macular data	PSP patients	Controls	p-values
Total macular volume (mm^3^)	7.9 ± 0.1	8.6 ± 0.04	<0.0001*
Circle center thickness C0 (µm)	264.9 ± 6.1	284.4 ± 2.1	0.003*
Center thickness (µm)	230.8 ± 6.0	237.5 ± 1.8	0.02*
Central Minimum (µm)	212.0 ± 2.1	234.7 ± 2.0	0.001*
Central Maximum (µm)	321.7 ± 7.3	331.8 ± 2.4	0.19
Central nasal thickness N1 (µm)	333.9 ± 4.2	340.6 ± 1.4	0.13
Peripheral nasal thickness N2 (µm)	302.2 ± 4.0	293.2 ± 1.3	0.03*
Central superior thickness S1 (µm)	331.0 ± 4.1	345.4 ± 1.4	0.001*
Peripheral superior thickness S2 (µm)	284.1 ± 4.1	295.1 ± 1.2	0.01*
Central temporal thickness T1 (µm)	318.8 ± 4.1	341.1 ± 1.4	<0.0001*
Peripheral temporal thickness T2 (µm)	273.5 ± 3.9	288.2 ± 1.3	0.01*
Central inferior thickness I1 (µm)	328.9 ± 4.1	341.6 ± 1.4	0.004*
Peripheral inferior thickness I2 (µm)	280.3 ± 4.1	285.8 ± 1.3	0.2

P-values with significant differences are marked with *.

Thickness reductions were found in the majority of the eight macular sectors in the PSP group (Table 3 and Fig. 2A): central sector C0 (264.9 ± 6.0 μm versus 284.3 ± 2.0 μm, p = 0.003), central temporal sector T1 (318.8 ± 4.1 μm versus 341.1 ± 1.4 μm, p < 0.001), central inferior sector I1 (328.9 ± 4.1 μm versus 341.5 ± 1.4 μm, p = 0.004), central superior sector (331.0 ± 4.1 μm versus 345.4 ± 1.4 μm, p = 0.001), peripheral superior sector S2 (284.1 ± 4.0 μm versus 295.1 ± 1.2 μm, p = 0.01), and peripheral temporal sector T2 (273.5 ± 3.9 μm versus 288.3 ± 1.3 μm). However, the peripheral nasal sector N2 showed a slightly thicker macular area in PSP patients compared to controls (302.2 ± 4.0 μm versus 293.2 ± 1.3 μm, p = 0.03).

Again, we did not find a significant correlation with disease duration or the PSP-RS.

### Scanning laser polarimetry (SLP)

**Figure 3:**
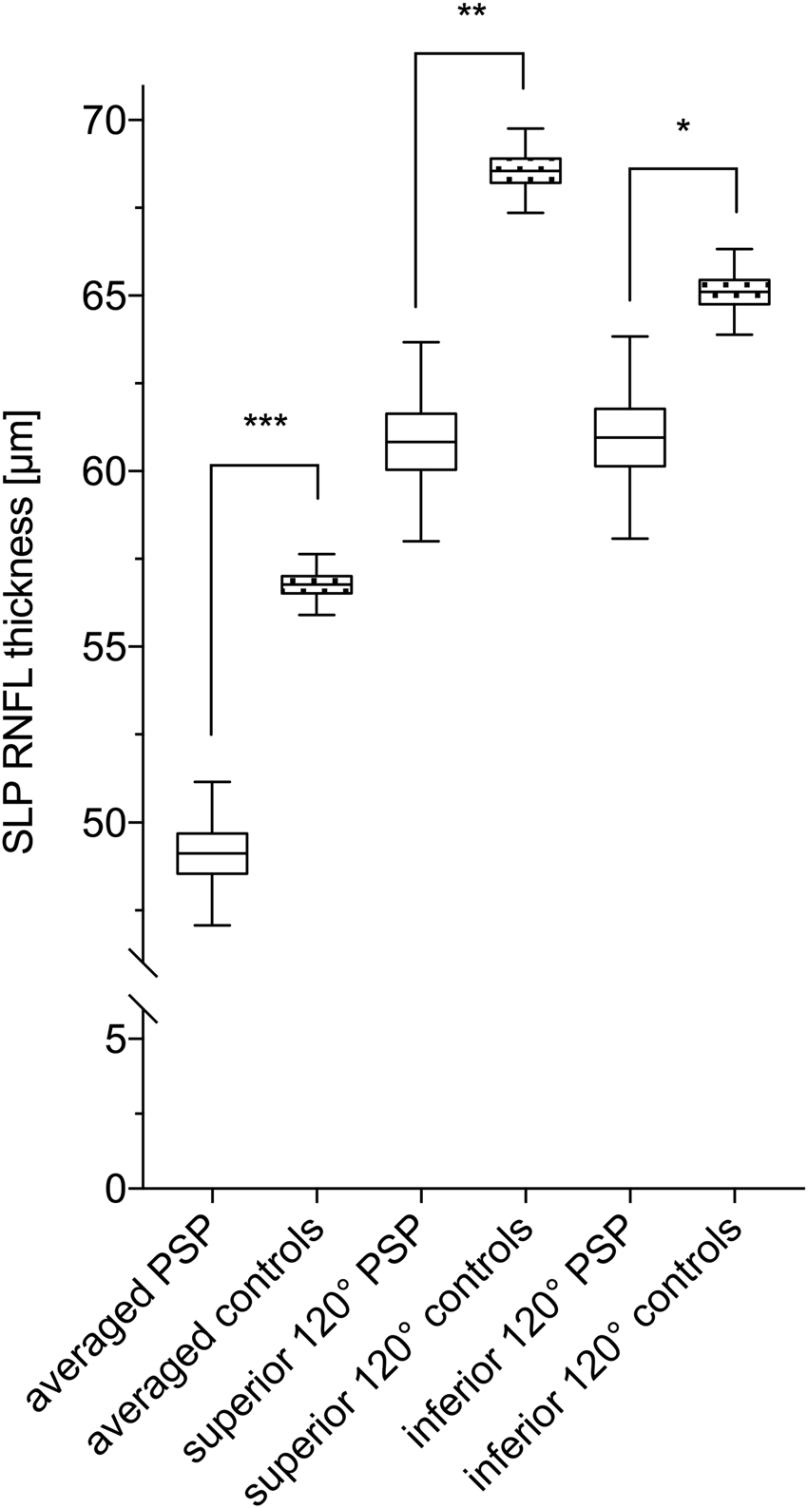
RNFL thickness data of PSP patients and controls measured by SLP is depicted. Significantly different sectors are shown. ns: not significant p > 0.5, *p ≤ 0.5, **p ≤ 0.01, ***p ≤ 0.001.

The average RNFL was significantly thinner in PSP patients than in the control group [49.1 ± 1.1 μm versus 56.8 ± 0.5 μm, p < 0.0001 (mean ± standard error)], as well as the superior 120° (60.8 ± 1.5 μm versus 68.6 ± 0.7 μm, p < 0.001) and the inferior 120° of the central retina around the optic nerve head (61.0 ± 1.6 μm versus 65.1 ± 0.7 μm, p = 0.02) (s. Fig. 3). The nerve fiber index (NFI) in the PSP group showed significantly larger values, which indicates more retinal degeneration (22.3 ± 1.6 versus 16.0 ± 0.7, p < 0.001).

Disease duration and the PSP-RS showed no correlation with the measured values.

### Comparison of OCT and SLP

**Figure 4:**
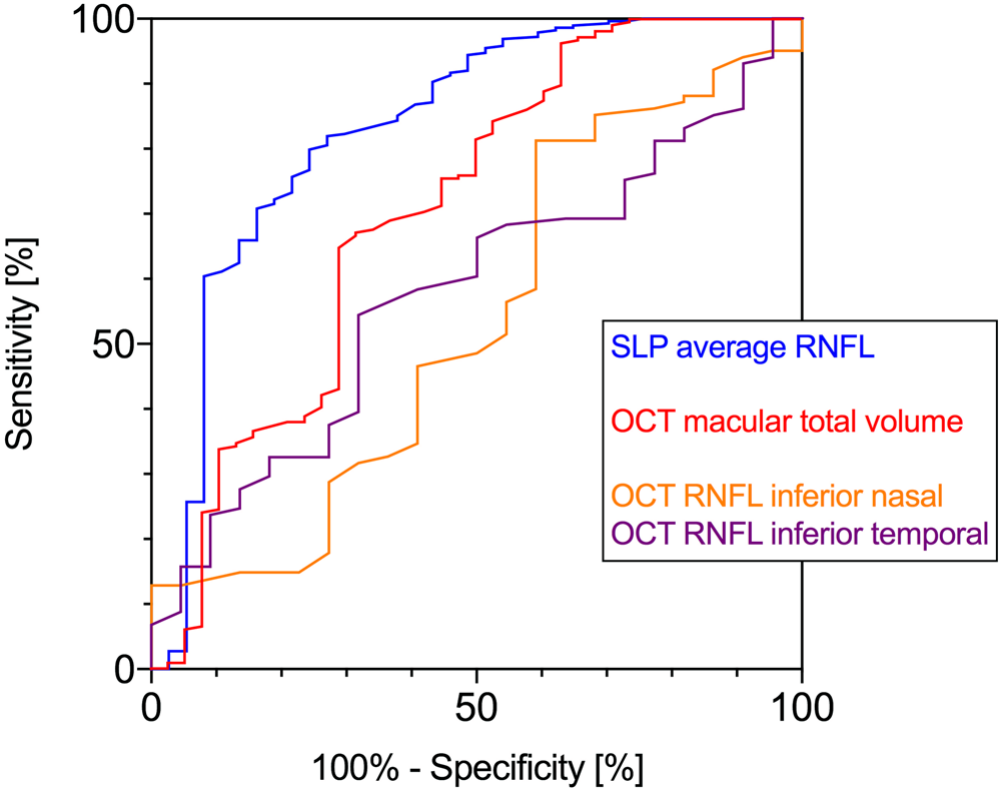
Receiver operating characteristic curves of SLP average RNFL thickness, OCT RNFL inferior nasal and OCT RNFL inferior temporal thickness and OCT total macular volume are plotted.

The area under the curve (AUC) of the SLP average RNFL (0.83 ± 0.04) was significantly larger than the AUC of the OCT RNFL inferior nasal (0.53 ± 0.07; p < 0.001) and the OCT RNFL inferior temporal thickness (0.58 ± 0.06; p < 0.001) (s. Fig. 4). The AUC of the SLP average RNFL was also larger than the OCT macular total volume (AUC: 0.70 ± 0.05; p = 0.055), but the difference was not significant. This indicates superior diagnostic validity of SLP compared to OCT in terms of RNFL measurement.

## Discussion

Applying two devices with different measuring techniques (SD-OCT and SLP), we found a reduced average RNFL thickness in the inferior nasal area and in the inferior temporal area around the optic nerve head, a reduction in the macular volume, and a thinning of many macular sectors, as well as the central minimum using SD-OCT in PSP patients compared to controls. The SLP revealed a decreased RNFL thickness of the superior and inferior 120° and an increase in the NFI in PSP patients compared to the control group. Retinal changes were independent of disease duration or the PSP-RS total score in any SD-OCT or SLP measurement. Diagnostic validity regarding sensitivity and specificity to discriminate PSP patients against controls was higher when applying SLP compared to SD-OCT.

It has been shown that the optic nerve and the retina, which embryologically originate from the diencephalon, are affected in several diseases of the CNS (such as multiple sclerosis and PD) where impairments such as visual acuity, color vision and contrast^4^, and diplopia^5^ can be found.

The retina contains dopaminergic cells^21, 22^. In animal models of PD, dependency of higher visual areas on dopamine have been shown^23^, which implies that dopamine deficits may influence the visual processing in patients with a dopaminergic deficit, such as PD or PSP. Furthermore, aggregation of the protein tau might play a role in retinal degeneration in PSP. Tau deposition has been found in adult human retinae^24^, and marked retinal degeneration is known in Alzheimer disease, another more common tauopathy^25, 26^. We found profound retinal changes in PSP patients compared to controls and compared to changes described for PD^7, 17^. This reflects clinical observations that describe oculomotor and visual impairment^27^ and brain atrophy^28–30^ in PSP as being distinct from PD or controls.

However, it is unknown whether retinal changes are related to degeneration within the retina or are due to central degenerative processes with “downstream effects”. Future studies should be applied to investigate these histo-pathophysiological relations.

As far as we know, this is the third study to use OCT and the first study to use SLP for examining patients with PSP and, furthermore, the first study to combine OCT and SLP analyses in these patients. Recently, OCT data of 40 patients with PD has been compared with data from patients with atypical parkinsonian syndromes such as multiple system atrophy (MSA, n = 19), progressive supranuclear palsy (PSP, n = 15), corticobasal syndrome (CBS, n = 10) and 35 controls^17^. Interestingly, the latter study, similar to our results, described a reduction in the thickness and volume of the paramacular areas, but no change in the mean RNFL (we found reductions in the inferior temporal and inferior nasal areas). By segmenting the retinal layers in the macular area, they detected a reduction in the complex of retinal ganglion cells and inner plexiform and outer nuclear layers in PSP, but not in MSA or CBS patients compared to controls. Using Cirrus OCT without an eye-tracking system, Schneider *et al*.^31^ found a reduction of the entire retinal thickness and of the inner nuclear layer in PSP patients compared to controls, as well as changes in the outer nuclear (thinner) and outer plexiform (thicker) layers compared to MSA patients.

We also detected a thinning of the total macular volume in PSP patients, consistent with Albrecht *et al*.^17^, and a reduced thickness of the majority of macular sectors. Degeneration of the foveal pit within the macula and the annular area around the fovea were already found in PD^12, 32, 33^. These areas might be sensitive locations for retinal changes associated with neurodegenerative diseases with dopaminergic deficit. Noteworthy is that the central vision of the fovea within the macula is important for gait and walking and for fast saccades, functions, which are especially severely impaired in PSP.

For the first time SLP was applied as a new and additional approach to assess retinal changes in PSP patients. Using SLP, we found a widespread alteration of the RNFL throughout the retina with thinning in the inferior retinal area (consistent with our OCT data) and the superior area and, thereby, an increase in the NFI compared to controls. Because SLP showed a higher sensitivity and specificity in discrimination of PSP patients and controls than SD-OCT, the combined use of both techniques improves the diagnostic quality of retinal imaging as a potential additional tool in PSP diagnosis.

### Clinical and scientific value of our findings

Our data suggests that retinal changes in PSP are independent of disease duration and severity. This might facilitate an earlier clinical diagnosis of PSP and potentially a differential diagnosis between PD and PSP, which is often difficult, especially in the first years of the disease course where the clinical picture can be inconclusive and morphological signs, such as alterations in MRI, are missing.

Applying SLP we found reductions in the RNFL thickness of PSP patients with even higher sensitive and specific power than using SD-OCT. Furthermore, in addition to our previous findings with SLP showing an increase in the NFI in patients with PD^7^, we found even higher values in patients with PSP in the present study, which is consistent with more degenerative changes in the CNS in PSP compared to PD.

The building of specific diagnostic formulas based on OCT results has been proposed by Garcia-Martin *et al*. as indicative for PD^11^. Thus, our method of using SLP in addition to SD-OCT and thus combining more than one value is supposed to improve the diagnostic sensitivity and specificity. Noteworthy, the NFI, which is calculated by a vector algorithm combining several RNFL values, showed a large difference between controls and patients with PD^7^ as well and was found significantly different between controls and PSP patients here. Because PSP patients in this study were compared to the nearly identical control group that was used in the study with PD patients^7^, we assume that the increase in the NFI in PSP is relatively larger than in Parkinson patients^7^. Therefore, SD-OCT and SLP might serve as supplementary diagnostic tools to detect early morphological retinal changes in PD, and even more distinctly in PSP patients. Furthermore, studying retinal changes in PSP could improve our insight into the degenerative processes of patients with PSP, especially when looking at single layer analysis^17, 31^.

### Study strengths and limitations

As described, a strength of the present study is the combined diagnostic application of SD-OCT and SLP in PSP patients and the comparison of data with a large normative database. As further beneficial aspect, the probability of PSP diagnoses was specified considering clinical data of longitudinal neurological follow-up examinations, response to dopaminergic treatment (both as short-term levodopa test and by levodopa response during clinical follow-up), diagnostic MRI, and in nine cases scintigraphy (FP-CIT-, IBZM- or MIBG-SPECT) (Table 1).

### Conclusion and Outlook

In PSP patients, retinal degeneration seems to be part of the neurodegenerative process and might lead to reduced visual acuity. Patients show atrophy of the total macular volume, changes of parafoveal areas, reductions of the central minimum, and thinning of the RNFL as measured by two devices. Retinal changes were measureable independent of disease severity and duration. With about 6.5 µm thinner average RNFL and about 2.5 µm thinner inferior sector of the RNFL in SLP measurements, changes were more profound in PSP than described by us for PD patients using the same device and nearly the identical group of controls^7^. The combined application of SD-OCT and SLP as a fast, non-invasive, and cost-efficient method to assess retinal morphology might be a complementary diagnostic tool in future for patients with movement disorders. In PSP, it might support early diagnosis when clinical symptoms and other supportive technical applications are still inconclusive; it might also facilitate discrimination between PD and other parkinsonian syndromes^17, 34^. However, larger scale studies, especially with a longitudinal approach, are needed to establish retinal imaging techniques as a diagnostic and potential therapeutic control instrument for patients with parkinsonian syndromes, including PSP.

## Methods

### Patients and controls

Twenty-two patients (10 females and 12 males, 66.2 ± 6.5 years, range 49–73 years) diagnosed with PSP were included in this prospective cross-sectional study. They were recruited from the movement disorder center of a university hospital between June 2015 and February 2016. The diagnosis of “clinically possible” or “clinically likely” PSP was made by a movement disorders specialist (CB or UH) according to the clinical criteria for the diagnosis of progressive supranuclear palsy set out by the National Institute for Neurological Disorders and Society for PSP (NINDS-SPSP)^20^. Disease severity was assessed and scored according to the progressive supranuclear palsy rating scale (PSP-RS)^35^. This scale comprises 28 items in six categories assessing daily activities (by history), behavior, bulbar and ocular motor signs, limb motor function, and gait/midline status. Achieved scores were added to a total score (0–100 possible). Disease duration was evaluated by disease onset, defined as occurrence of first symptoms according to medical history. The data of PSP patients were compared with the data of 151 healthy controls who had been sourced from our database and who had sufficient OCT and SLP data. Patients and controls signed informed consent before entering the study. The research protocol considered the Good Clinical Practice (GCP) criteria, followed the recommendations of the Declaration of Helsinki (7^th^ revision, 64^th^ meeting, Fortaleza, Brazil), and was approved by the local ethics committee of the Hamburg Medical Council.

After confirmation of the diagnosis and assessment of the PSP-RS, patients and controls were examined by an ophthalmic specialist (BS or RK) to exclude ophthalmic disorders that would interfere with the measurements. Patients with maculopathies (e.g. age-related macular degeneration, epiretinal gliosis, macular edema), glaucoma, uveitis, diabetic retinopathy, or high myopia (>5 diopters) were excluded. After best corrected visual acuity assessment, intraocular pressure was measured by non-contact tonometry (Nidek Tonometer NT-530, Nidek, Japan) to exclude non-detected glaucoma, and the anterior and posterior segments were assessed by slit-lamp biomicroscopy with non-dilated pupils. Eyes of patients or controls with other reasons for a reduced visual acuity e.g. a significant cataract were excluded from the analysis. Patients and controls then underwent SD-OCT image acquisition (SPECTRALIS; Heidelberg Engineering, Heidelberg, Germany, software version 1.9.10.0, acquisition module version 6.3.2) and SLP examination (GDx ECC, Carl Zeiss Meditec, version 1.1.1, Dublin, USA).

### Optical coherence tomography (OCT)

**Figure 5:**
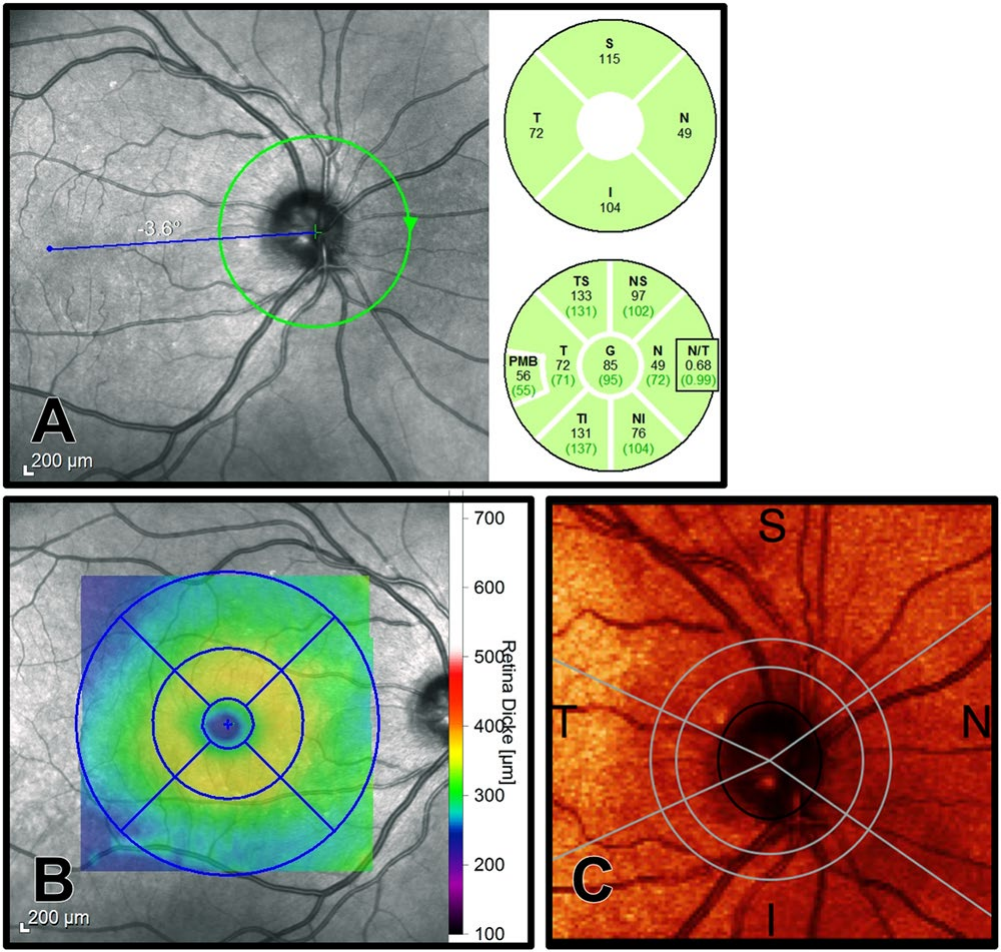
Example of OCT and SLP images. (**A**) Circular OCT RNFL scan around the optic nerve head divided into eight defined sectors. (**B**) Perifoveal volumetric OCT scan measuring the total macular volume and the thickness of the central retina within the central, inner and outer ring in four quadrants each. (**C**) SLP peripapillary scan measuring average RNFL thickness, superior and inferior thickness, and the proprietary nerve fiber index (NFI).

For measuring the RNFL, a 3.4 mm ring scan was placed around the optic disc and analyzed with the RNFL-Nsite software tool. This divides the RNFL into eight defined sectors (Fig. 5A): (1) temporal quadrant, (2) superior temporal quadrant, (3) superior quadrant, (4) superior nasal quadrant, (5) nasal quadrant, (6) inferior nasal quadrant, (7) inferior quadrant, and (8) inferior temporal quadrant. The software also computes the RNFL thickness of the papillomacular bundle (PMB) and the ratio of nasal versus temporal RNFL thickness (N/T ratio).

For the macular area, a perifoveal volumetric scan consisting of 61 B-Scans was performed to measure the total macular volume and the thickness of the central retina. A circle with 6-mm diameter was centered at the fovea with a central 1-mm disc and inner and outer rings of 3 and 6 mm (see Fig. 5B). These rings were divided into four quadrants, resulting in eight sections (inner superior thickness, inner nasal thickness, inner inferior thickness, inner temporal thickness, outer superior thickness, outer nasal thickness, outer inferior thickness, and outer temporal thickness). The central minimum and maximum thicknesses (within this macular scan) were calculated as well.

Scans were controlled for scan quality; scans with a quality < 15 dB were excluded. No manual corrections were applied, and scans with segmentation errors were not used in the analysis.

Data was correlated with disease duration and the global value of the PSP-RS.

### Scanning laser polarimetry (SLP)

We applied SLP with enhanced corneal compensation (Glaucoma Diagnostics, GDx ECC, Carl Zeiss Meditec, Dublin, USA) and compared this data with the SD-OCT results. The SLP scans were centered at the optic disc and performed in distances of 2.4 and 3.2 mm (bandwidth 0.4 mm). Scans with a quality below six were excluded. The average RNFL thickness (TSNIT average), superior thickness (superior 120°), inferior thickness (inferior 120°) and the nerve fiber index (NFI) were determined from the measured data (see Fig. 5C). The NFI was calculated by the device analyzing the whole RNFL thickness data (values 0–100; higher values show more thinning of the RNFL).

Data was correlated with disease duration and the global value of the PSP-RS.

### Statistics

Statistical evaluations were performed using SPSS software (version 22.0, SPSS Inc., Chicago, USA) and GraphPad Prism software (version 7.0a, GraphPad Software Inc., La Jolla, USA). After adjusting the data for age and sex differences between the two groups (patients and controls), data was compared using a multivariate analysis with mixed models. Visual acuity in LogMAR was compared using a parametric t-test with two-tailed p-values. We decided to use the large healthy control group to improve the quality of the normative database. Both eyes of each proband were included in the analysis whenever possible, so it was corrected for repeated measuring.

In order to compare the discriminatory power of the applied different imaging methods to detect PSP patients we calculated receiver operating characteristic (ROC) curves and compared the area under the curve (AUC). For each technique, the RNFL data with the smallest p-value was selected: SLP average RNFL, OCT RNFL inferior nasal and OCT RNFL inferior temporal thickness and the macular parameter of the OCT total macular volume (s. Fig. 4). Each sensitivity is the fraction of values in the patient group that were above the threshold. The specificity is the fraction of values in the control group that were below the threshold. Confidence intervals were computed using the observed proportion by the Clopper method^36^, without correction for multiple comparisons. To compare the power of the selected methods we used a two-tailed t-test for comparing the AUC. P-values of 0.05 or smaller were considered to show significance.
